# Bioinformatics analysis of the role of aldolase A in tumor prognosis and immunity

**DOI:** 10.1038/s41598-022-15866-4

**Published:** 2022-07-08

**Authors:** Wanjia Tian, Junying Zhou, Mengyu Chen, Luojie Qiu, Yike Li, Weiwei Zhang, Ruixia Guo, Ningjing Lei, Lei Chang

**Affiliations:** 1 Department of Obstetrics and Gynecology, The First Affiliated Hospital of Zhengzhou University, Zhengzhou University, Zhengzhou, 450000 Henan China; 2 Academy of Medical Sciences of Zhengzhou University, Zhengzhou University, Zhengzhou, 450000 Henan China; 3 School of Basic Medical Sciences, Zhengzhou University, Zhengzhou, 450000 Henan China

**Keywords:** Cancer, Computational biology and bioinformatics

## Abstract

Aldolase A (ALDOA) is an enzyme that plays an important role in glycolysis and gluconeogenesis, which is closely related to tumor metabolism. In this study, the overall roles of ALDOA in pan-cancer have been investigated from several aspects using databases and online analysis tools. Using the ONCOMINE database, the expression of ALDOA in various cancers was analyzed. The prognostic role of ALDOA was explored by PrognoScan, GEPIA, and Kaplan–Meier Plotter. The immune-related role of ALDOA and its downstream substrates was decided by TIMER, cBioPortal and String. Our data indicate that ALDOA expression level in lung adenocarcinoma, liver hepatocellular carcinoma, head and neck squamous cell carcinoma is higher than that in normal tissues. Increased expression of ALDOA often indicates a poor prognosis for patients. The correlation between ALDOA and immune infiltration among different tumors is very different. We also investigate the relationship between ALDOA and its upstream/downstream proteins. Our results showed that ALDOA could be used as a biomarker for the tumor prognosis, and could be correlated with the infiltrating levels of macrophages, CD4+ T cells and CD8+ T cells.

## Introduction

Glucose metabolism is the main way for cells to obtain energy, and glycolysis is the main way for the body to obtain energy when the body is relatively hypoxic^1^. But the Warburg effect and a large number of studies have shown that, unlike normal cells, the energy production of tumor cells depends on glycolysis^2,3^. The extracellular matrix (ECM) remodeling of tumor cells promotes high-speed glucose metabolism, and the active glycolytic activity of tumor cells can limit the activation of T cells and promote tumor progression^4,5^. Therefore, enzyme inhibitors in glycolysis are a potential anti-cancer research program pursued by many researchers.

Aldolase is a key enzyme in glycolysis, which can be divided into three types: ALDOA, ALDOB, and ALDOC^6^. Aldolase A (ALDOA) plays an important role in the regulation of cell shape and mobility, striated muscle contraction, actin filament organization and ATP biosynthesis^6^. In addition, ALDOA is one of the most abundant glycolytic enzymes in tumor cells^7^. A number of studies have identified roles of ALDOA in promoting tumor growth and metastasis in hepatocellular carcinoma, cervical adenocarcinoma, osteosarcoma, pancreatic cancer, lung cancer and other tumors^8–12^. Cumulatively, studies on liver cancer and lung cancer are the most abundant. Phosphorylation of ALDOA can enhance the glucose metabolism of liver cancer cells, thereby promoting their growth and tumor formation^13^. Some studies also verify that high expression of ALDOA predicts poor prognosis for patients^14,15^. However, there is no clear report on the role of ALDOA in pan-cancer.

Proliferating cancer cells will also change the metabolic components of the ECM around them, of which the accumulation of lactic acid is the most common^16^. The increase in lactic acid levels is closely related to the formation of the immune microenvironment^17–19^. Recent research focuses a lot on understanding the tumor immune microenvironment^20^. It has been found that tumor infiltrating immune cells can promote tumor cell survival and proliferation, which can also provide signals for tumor immunosuppression^21^. But there have also been studies showing that tumor-infiltrating B cells play an anti-tumor role in lung cancer^22^. Stromal cells in the microenvironments are also important. For example, cancer associated fibroblasts (CAFs), as one of the main cell populations in the tumor microenvironment (TME), play an indispensable role in the progression of tumors^23^. Therefore, whether ALDOA has any immune-related functions or it is correlated with the infiltration of immune cells in tumors should be explored.

In this project, we used the TCGA project and GEO databases to perform a pan-cancer analysis on ALDOA for the first time. We analyze the pathogenesis and clinical prognostic value of ALDOA in different tumors based on a series of visual results, such as gene expression, prognosis analysis, immune infiltration, immune correlation, and co-expression of genes and proteins. The results of this study may provide comprehensive information of ALDOA in predicting the prognosis of tumor patients, and further investigation in the tumor immune microenvironment.

## Results

### The mRNA expression of ALDOA in pan-cancer

**Figure 1:**
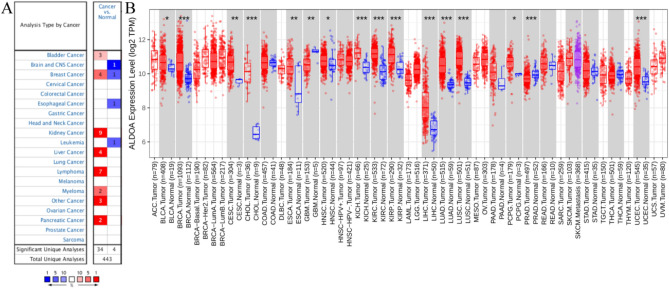
The expression of ALDOA in different tumors. (**A**) Compared with the normal tissues in ONCOMINE (www.oncomine.org), the expression changes of ALDOA in different cancer tissues. The number in each cell is the number of data sets. (**B**) Used TIMER 2.0 (http://timer.cistrome.org/) to compare the expression of ALDOA in different tumors in the TCGA database. *P < 0.05; **P < 0.01; ***P < 0.001.

Since ALDOA is reported in several cancer studies, we first analyzed the overall expression levels of ALDOA mRNA in different tumors compared to normal by Oncomine. The results showed that ALDOA was highly expressed in bladder cancer, breast cancer, kidney cancer, liver cancer, lymphoma, myeloma, and pancreatic cancer, and low in brain and central nervous system (CNS) cancer, esophageal cancer, and leukemia. But there was a low expression in the 5 breast cancer data sets (Fig. 1A). To further evaluate the expression of ALDOA in tumors, we used TIMER2 to detect TCGA RNA sequencing. These results were shown in Fig. 1B. Compared with normal tissues, ALDOA was under-expressed in Glioblastoma multiforme (GBM). However, ALDOA was highly expressed in BLCA (bladder urothelial carcinoma), BRCA (breast invasive carcinoma), CESC (cervical squamous cell carcinoma and endocervical adenocarcinoma), CHOL (cholangiocarcinoma), ESCA (esophageal carcinoma), HNSC (head and neck squamous cell carcinoma) , KICH (kidney chromophobe), KIRC (kidney renal clear cell carcinoma), KIRP (kidney renal papillary cell carcinoma), LIHC (liver hepatocellular carcinoma), LUAD (lung adenocarcinoma), LUSC (lung squamous cell carcinoma), PCPG (pheochromocytoma and paraganglioma), PRAD (prostate adenocarcinoma) and UCEC (uterine corpus endometrial carcinoma) compared to normal tissues.

### Prognostic analysis of ALDOA in pan-cancer

**Figure 2:**
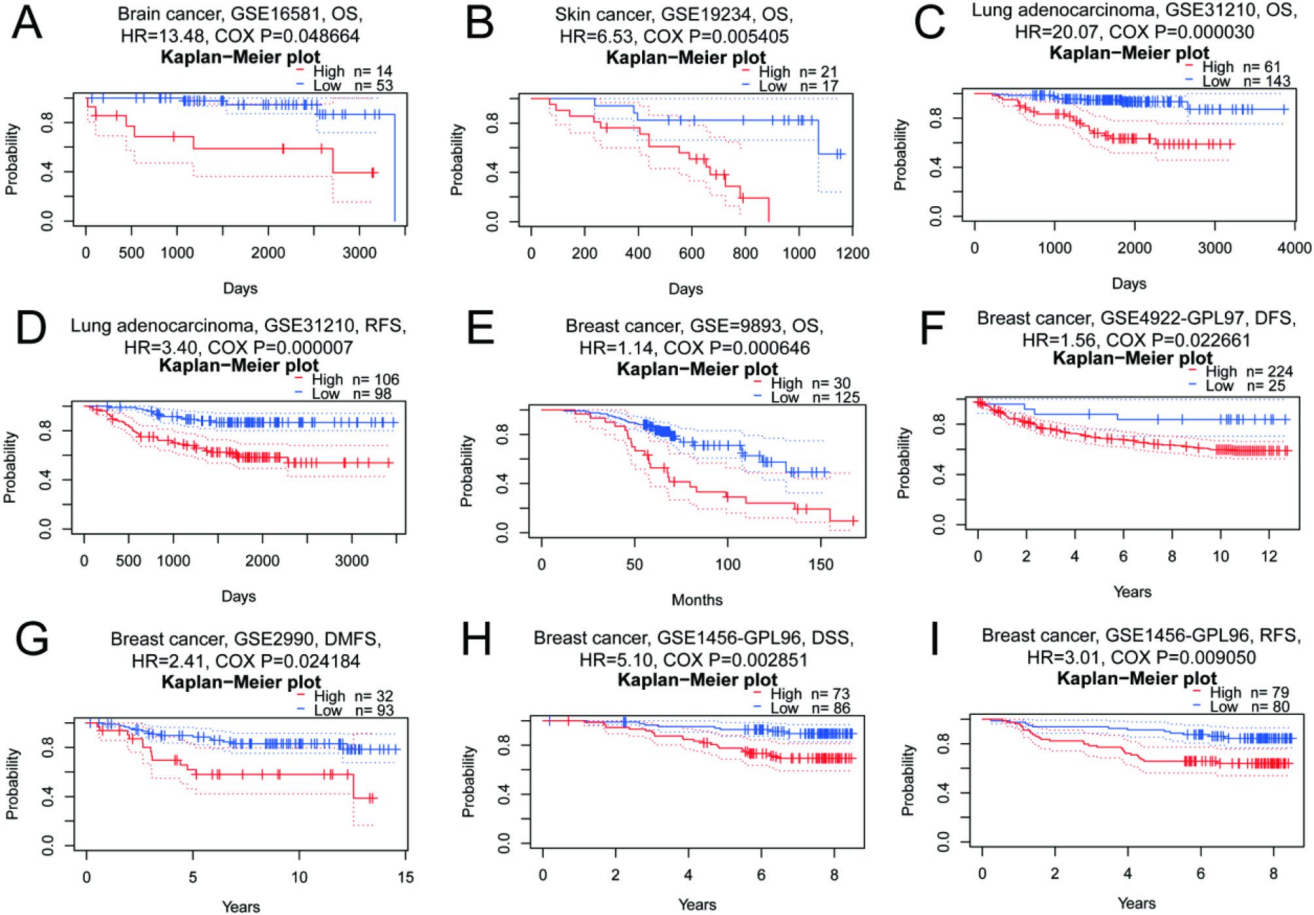
The influence of the expression level of ALDOA in different tumors on the survival curve in PrognoScan (http://dna00.bio.kyutech.ac.jp/PrognoScan/index.html). (**A**) OS (n = 67) in brain cancer cohort GSE16581. (**B**) OS (n = 38) in skin cancer cohort GSE19234. (**C**,**D**) OS (n = 204) and RFS (n = 204) in lung adenocarcinoma cohort GSE31210. (**E**) OS (n = 155) in breast cancer cohort GSE9893. (**F**) DFS (n = 249) in breast cancer cohort GSE4922-GPL97. (**G**) DMFS (n = 125) in breast cancer cohort GSE2990. (**H**,**I**) DSS (n = 159) and RFS (n = 159) in breast cancer cohort GSE1456-GPL96. OS, overall survival; RFS, relapse-free survival; DFS, disease-free survival; DMFS, distant metastasis-free survival; DSS, disease-specific survival; RFS, relapse-free survival.

Next, we used different databases to analyze the prognostic value of ALDOA in pan-cancer. Prior to prognostic analysis, the data set was analyzed. We tested the Proportional Hazards assumption of a Cox Regression using Schoenfeld residuals (Supplementary Fig. 1 and 2). If the test of proportional hazards (PH) assumption indicated to refuse the assumption (as there is a significant relationship between the residuals and time, *P* < 0.05), accelerated failure time (AFT) model was applied to further evaluate the association between the outcome and the expression of ALDOA (Supplementary Table 1). According to our result, the expression level of ALDOA was significantly related to the prognosis of brain cancer, skin cancer, lung adenocarcinoma and breast cancer in the PrognoScan database (Fig. 2). Highly expressed ALDOA leads to poor prognosis of these four tumors, including brain cancer (OS: total number = 67, Hazard Ratio, HR = 13.48, Cox *P* = 0.048664), skin cancer (OS: total number = 38, HR = 6.53, Cox *P* = 0.005405), lung adenocarcinoma (OS: total number = 204, HR = 20.07, Cox *P* = 0.000030; RFS: total number = 204, HR = 3.40, Cox *P* = 0.000007) and breast cancer (OS: total number = 155, HR = 1.14, Cox *P* = 0.000646; DFS: total number = 249, HR = 1.56, Cox *P* = 0.022661; DMFS: total number = 125, HR = 2.41,Cox *P* = 0.024184; DSS: total number = 159, HR = 5.10, Cox *P* = 0.002851; RFS: total number = 159, HR = 3.01, Cox *P* = 0.009050).

**Figure 3:**
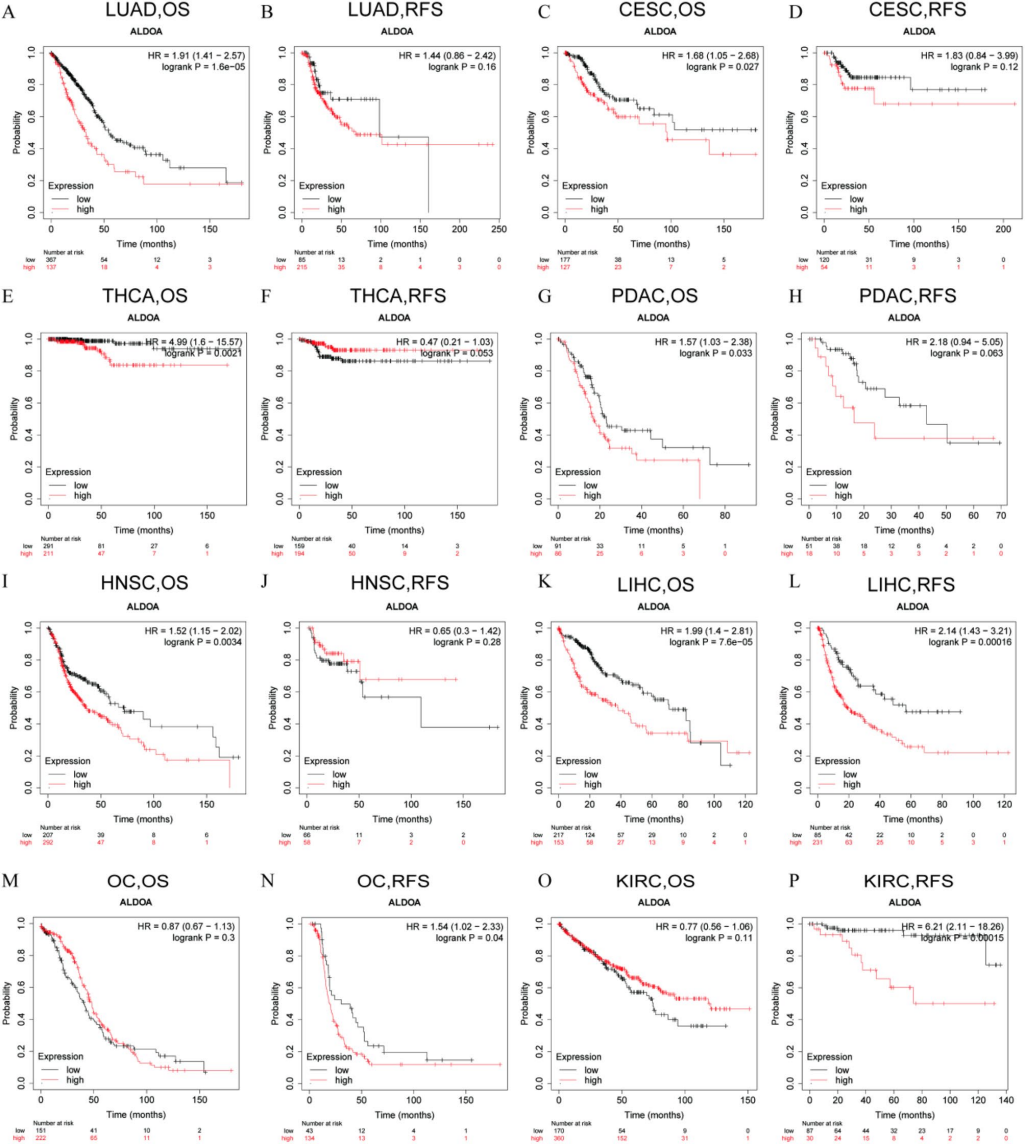
The influence of the expression level of ALDOA in different types of cancer in Kaplan–Meier Plotter (https://kmplot.com/analysis/) on the survival curve. OS and RFS of (**A**,**B**) lung adenocarcinoma (LUAD) (**C**,**D**) cervical squamous cell carcinoma and endocervical adenocarcinoma (CESC) (**E**,**F**) thyroid carcinoma (THCA) (**G**,**H**) pancreatic ductal adenocarcinoma (PDAC) (**I**,**J**) head and neck squamous cell carcinoma (HNSC) (**K**,**L**) liver hepatocellular carcinoma (LIHC) (**M**,**N**) ovarian cancer (OC), and (**O**,**P**) kidney renal clear cell carcinoma (KIRC). Red curve represents patients with high expression of ALDOA.

Since the data in PrognoScan came from the gene expression omnibus (GEO) database, we used Kaplan–Meier Plotter, which is based on the TCGA database for further survival (OS and RFS) verification. After we excluded the intersected curves, we found that, consistent with the above data, the high expression of ALDOA in LUAD affected the OS of patients (LUAD: HR = 1.91, 95% CI from 1.41 to 2.57, logrank *P* = 1.6e−05) (Fig. 3A). For LUAD, CESC, THCA, PDAC and HNSC, the expression level of ALDOA significantly affected the OS of patients (LUAD: OS, HR = 1.91, 95% CI from 1.41 to 2.57, logrank *P* = 1.6e−05; CESC: OS, HR = 1.68, 95% CI from 1.05 to 2.68, logrank *P* = 0.027; THCA: OS, HR = 4.99, 95% CI from 1.6 to 15.57, logrank *P* = 0.0021; PDAC: OS, HR = 1.57, 95% CI from 1.03 to 2.38, logrank *P* = 0.033; HNSC: OS, HR = 1.52, 95% CI from 1.15 to 2.02, logrank *P* = 0.0034) but not RFS (Fig. 3A–J). On the contrary, for LIHC, OC and KIRC, the expression level of ALDOA was related to RFS (LIHC: RFS, HR = 2.14, 95% CI from 1.43 to 3.21, logrank *P* = 0.00016; KIRC: RFS, HR = 6.21, 95% CI from 2.11 to 18.26, logrank *P* = 0.00015) but not to OS (SARC: OS, HR = 0.68, 95% CI from 0.45 to 1.04, logrank *P* = 0.071; OC: OS, HR = 0.87, 95% CI from 0.67 to 1.13, logrank *P* = 0.3; KIRC: OS, HR = 0.77, 95% CI from 0.56 to 1.06, logrank *P* = 0.11) but not OS (Fig. 3K–P).

In addition, the RNA expression level of ALDOA of different tumors in TCGA was analyzed by GEPIA2. We found that ALDOA mainly affected the OS in the pan-cancer rather than DFS (OS: HR = 1.3, logrank P = 7.5e−15; DFS: HR = 1.1, logrank P = 0.079) (Supplementary Fig. 3A). High expression of ALDOA often indicated poor overall survival in SKCM, THCA, THYM and PAAD. However, ALDOA is associated with DFS in PRAD (Supplementary Fig. 3). All the above results indicate that there is a certain relationship between the expression of ALDOA and the prognosis of tumor.

### Analysis of the correlation between ALDOA and tumor immunity

**Figure 4:**
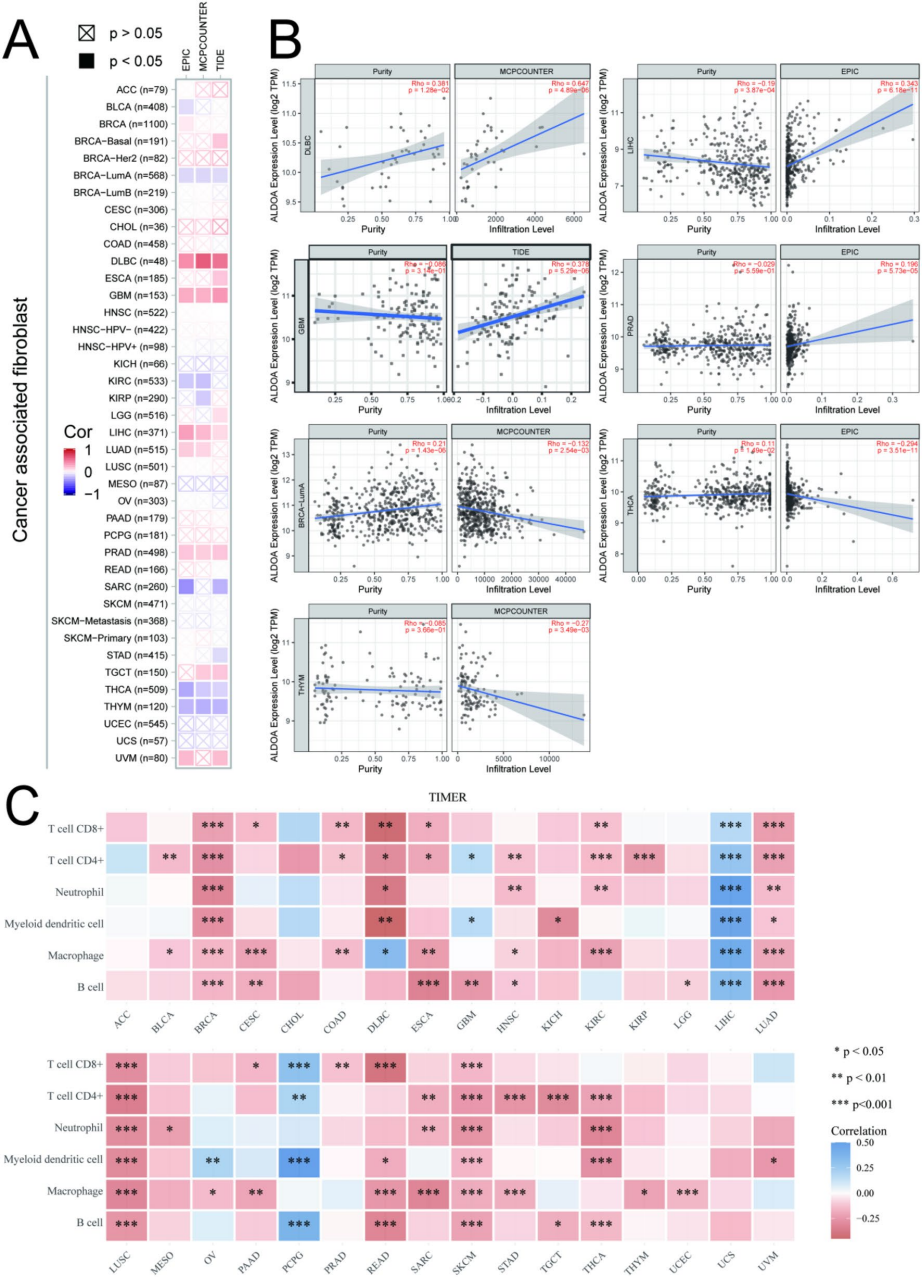
Correlation analysis between the expression level of ALDOA and the level of immune infiltration of cancer-related fibroblasts and immune cells by TIMER 2.0 (http://timer.cistrome.org/database). (**A**) Pearson correlation analysis between the expression level of ALDOA gene and the level of cancer-related fibroblast infiltration in pan-cancer; (**B**) Correlation between ALDOA expression level and tumor purity and immune infiltration level; (**C**) Spearman correlation analysis heat map of immune score and ALDOA gene expression in multiple tumor tissues, where the horizontal axis represents different tumor tissues, the vertical axis represents different immune scores, different colors represent correlation coefficients, and negative values represent negative correlations. A positive value represents a positive correlation. The stronger the correlation, the darker the color, *p < 0.05, **p < 0.01, ***p < 0.001), and the asterisk represents the importance (*p).

Next, we explore the relationship of ALDOA with stromal cells and infiltrated immune cells in the TME. We have observed that the expression of ALDOA in DLBC (lymphoid neoplasm diffuse large B-cell lymphoma), GBM, LIHC and PRAD is positively correlated with the infiltration of CAFs and negatively correlated with BRCA-lumA, THCA and THYM (thymoma) (Fig. 4A,B). We further evaluated the correlation between the expression of ALDOA and tumor immune cell infiltration in pan-cancer (Fig. 4C). In BRCA, LUSC and SKCM (skin cutaneous melanoma), this relationship is obviously negatively correlated, while in LIHC it is positively correlated.

In recent years, research on immune checkpoints and tumor immunotherapy has continued to progress^24^. Although PD1 (Programmed Cell Death Protein 1) and CTLA4 (Cytotoxic T-Lymphocyte Associated Antigen 4) are among the best, there are still other immune checkpoints under constant research^25^. Therefore, we selected LIHC and BRCA tumors and expanded 30 immune checkpoints to predict their prognostic relationship using Kaplan–Meier Plotter based on the TCGA database (Supplementary Fig. 4). We found that TMIGD2, CD27 and CD40LG have the effect of OS and RFS on LIHC and BRCA (Table 1). In addition, CD274, HHLA2, ICOS, BTLA, TNFRSF18, TNFSF4, HAVCR2 and NT5E all have an impact on the OS of LIHC and BRCA. Next, we studied the co-expression relationship between ALDOA and immune checkpoints in BRCA, LUAD, and SKCM (Supplementary Table 2). We found that in these three tumors, ALDOA and TNFSF4 are co-expressed (Table 2).

**Table 1:** Prognostic summary of the immune checkpoints in LIHC and BRCA.

		LIHC				BRCA			
		OS		RFS		OS		RFS	
		logrank *P*	HR	logrank *P*	HR	logrank *P*	HR	logrank *P*	HR
B7-CD28 family	CD274	0.042	0.7	0.39	0.85	0.0017	0.6	0.0092	0.57
	CD276	0.0018	1.74	0.19	1.25	0.1	1.33	0.017	1.8
	CTLA4	0.2	1.25	0.096	0.74	0.044	0.72	0.27	0.78
	HHLA2	0.00018	1.93	0.19	1.24	0.028	0.7	0.089	0.68
	ICOS	0.049	0.71	0.074	0.71	0.029	0.7	0.073	0.67
	ICOSLG	0.21	0.77	0.17	1.26	0.025	1.43	0.0001	2.29
	PDCD1	0.026	0.66	0.007	0.64	0.0031	0.6	0.1	0.69
	PDCD1LG2	0.028	0.66	0.0036	0.62	0.18	0.77	0.12	0.67
	TMIGD2	0.012	0.64	0.00084	0.57	0.00078	0.58	0.0028	0.52
	VTCN1	0.095	1.37	0.16	1.28	0.5	0.88	0.0084	0.56
The TNF superfamily	BTLA	0.001	0.56	0.00099	0.56	0.0016	0.6	0.054	0.6
	CD27	0.036	0.65	0.01	0.63	0.0032	0.61	0.035	0.63
	CD40	0.0077	1.63	0.035	1.45	0.064	0.72	0.16	0.7
	CD40LG	0.021	0.67	0.0032	0.56	3.20E−05	0.51	0.037	0.64
	CD70	0.27	0.8	0.0002	0.51	0.13	0.76	0.025	1.64
	TNFRSF18	0.035	1.45	0.35	1.18	0.0012	0.59	0.19	0.74
	TNFRSF4	0.00053	1.95	0.0074	1.6	0.23	0.81	0.019	1.69
	TNFRSF9	0.26	1.22	0.066	0.71	0.11	0.73	0.28	0.77
	TNFSF14	0.11	0.75	0.016	0.65	0.0037	0.63	0.11	0.69
	TNFSF4	0.0016	1.75	0.069	0.73	0.019	1.46	0.0072	1.8
	TNFSF9	0.081	1.4	0.15	0.77	0.22	1.24	0.0071	2.08
Others	ENTPD1	0.063	1.4	0.18	1.25	0.4	1.17	0.29	0.78
	FGL1	0.17	1.33	0.15	0.78	0.013	0.66	0.32	1.26
	HAVCR2	0.048	1.5	0.08	0.74	0.024	0.65	0.075	0.62
	IDO1	0.23	1.24	0.1	0.73	0.027	0.69	0.024	0.59
	LAG3	0.088	0.69	0.054	0.72	0.098	0.76	0.041	1.76
	NCR3	0.029	0.67	0.0035	0.6	0.08	0.64	0.0025	0.46
	NT5E	0.012	1.63	0.21	1.27	0.027	1.44	0.096	1.44
	SIGLEC15	0.098	0.74	0.0016	0.58	0.0043	0.63	0.039	0.6
	VSIR	0.19	1.26	0.07	0.71	0.12	0.77	0.016	0.59

**Table 2:** Mutual-exclusivity analysis between ALDOA and multiple-immune checkpoints in BRCA, LUAD and SKCM.

	A	B	Neither	A Not B	B Not A	Both	Log2 Odds Ratio	*p*-value	*q*-value	Tendency
BRCA	ALDOA	IDO1	3895	145	334	37	1.573	< 0.001	< 0.001	Co-occurrence
	ALDOA	VSIR	4161	168	68	14	2.35	< 0.001	< 0.001	Co-occurrence
	ALDOA	TNFSF4	3932	154	297	28	1.267	< 0.001	< 0.001	Co-occurrence
LUAD	ALDOA	TNFSF4	1394	17	74	6	2.733	< 0.001	0.014	Co-occurrence
	ALDOA	VTCN1	1434	19	34	4	> 3	0.002	0.025	Co-occurrence
SKCM	ALDOA	NCR3	1453	11	69	9	> 3	< 0.001	< 0.001	Co-occurrence
	ALDOA	CD27	1494	14	28	6	> 3	< 0.001	< 0.001	Co-occurrence
	ALDOA	NT5E	1474	14	48	6	> 3	< 0.001	< 0.001	Co-occurrence
	ALDOA	CD276	1469	14	53	6	> 3	< 0.001	< 0.001	Co-occurrence
	ALDOA	CD40LG	1510	17	12	3	> 3	< 0.001	0.006	Co-occurrence
	ALDOA	HHLA2	1425	14	97	6	2.654	0.001	0.01	Co-occurrence
	ALDOA	ICOSLG	1505	17	17	3	> 3	0.002	0.012	Co-occurrence
	ALDOA	TNFRSF4	1482	16	40	4	> 3	0.002	0.012	Co-occurrence
	ALDOA	TNFSF4	1481	16	41	4	> 3	0.002	0.013	Co-occurrence
	ALDOA	FGL1	1481	16	41	4	> 3	0.002	0.013	Co-occurrence
	ALDOA	TNFRSF9	1478	16	44	4	> 3	0.003	0.015	Co-occurrence
	ALDOA	VTCN1	1477	16	45	4	> 3	0.003	0.015	Co-occurrence
	ALDOA	TNFRSF18	1477	16	45	4	> 3	0.003	0.015	Co-occurrence
	ALDOA	CTLA4	1501	17	21	3	> 3	0.003	0.016	Co-occurrence
	ALDOA	ENTPD1	1466	16	56	4	2.71	0.006	0.026	Co-occurrence
	ALDOA	PDCD1	1459	16	63	4	2.533	0.009	0.036	Co-occurrence
	ALDOA	TMIGD2	1488	17	34	3	2.949	0.011	0.04	Co-occurrence

### ALDOA related gene and protein analysis

**Figure 5:**
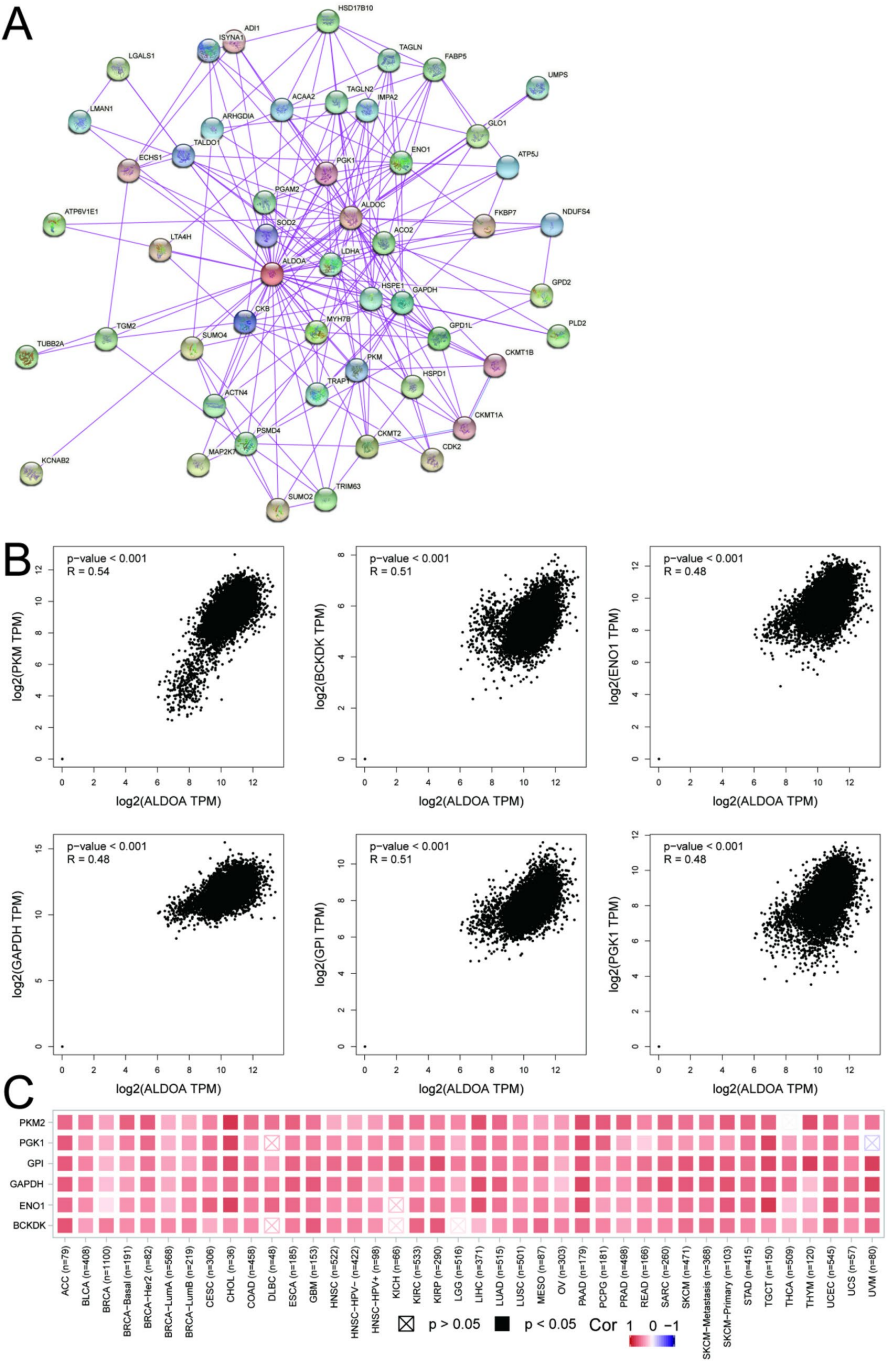
ALDOA related target gene analysis. (**A**) Proteins that have been experimentally determined to bind to ALDOA in the String (https://www.string-db.org/) database; (**B**) We searched the top 100 related genes related to ALDOA in the TCGA project through GEPIA2 (http://gepia2.cancer-pku.cn), and analyzed their expression correlation, including (PKM, BCKDK, ENO1, GAPDH, GPI and PGK1); (**C**) Co-expression of ALDOA and target genes in different tumor types in TIMER 2.0 (http://timer.cistrome.org/database).

Protein as the expresser of genetic information is closely related to the life activity characteristics of cells. Figure 5A shows the interaction network of 50 ALDOA binding proteins that have been experimentally confirmed based on the STRING tool. We collected 100 genes related to ALDOA expression in pan-cancer using GEPIA2, and obtained the first 6 most closely related genes. As shown in Fig. 5B, the expression level of ALDOA is positively correlated with PKM (pyruvate kinase M1/2) (R = 0.54), BCKDK (branched chain keto acid dehydrogenase kinase) (R = 0.51), ENO1 (enolase 1) (R = 0.48), GAPDH (glyceraldehyde-3-phosphate dehydrogenase) (R = 0.48), GPI (glucose-6-phosphate isomerase) (R = 0.51) and PGK1 (phosphoglycerate kinase 1) (R = 0.48). Corresponding to this, the heatmap also showed that the above six genes are positively correlated with ALDOA in most tumors (Fig. 5C).

## Discussion

The energy metabolism of tumors has its unique characteristics, such as increased glucose metabolism and increased lactate production^26^. In order to explore the role of ALDOA-regulated glycolysis in this change, we conducted a comprehensive analysis of ALDOA genes in 33 different tumors based on data from TCGA and GEO databases, prognostic effects, immune correlation, and related gene and protein analysis.

Most of the research on ALDOA focuses on liver cancer and lung cancer. For liver diseases, the high expression of ALDOA in patients with liver cirrhosis is closely related to the risk of liver cancer^27^. ALDOA has been proven to be an important regulator of the growth and progression of liver cancer cells under hypoxic conditions^8,28^. And ALDOA is also related to the prognosis of liver cancer patients^29^. This is consistent with our results. At the same time, we propose that this poor prognosis may be caused by the influence of ALDOA on immune infiltration.

For lung cancer, our results indicate that ALDOA affects the OS of LUAD rather than LUSC, which is consistent with the results of Wang Zhihao et al.^15^. However, the opposite result was shown in the immune-related screening, so we suspect that this result may be related to the level of infiltration of CAFs (Fig. 4A). But some studies have shown that ALDOA can promote the progression of lung squamous cell carcinoma and affect the prognosis^30^, which contradicts our results. This difference in results may be related to the sample size. At the same time, ALDOA has been proven to regulate the progression and metastasis of lung cancer through a variety of ways^12,31–33^. Therefore, the regulatory role of ALDOA in lung cancer and the prediction of prognosis are obvious.

For other tumors, ALDOA can also be used as an important biomarker for monitoring progress and predicting prognosis^10,34–37^. ALDOA plays the role as an oncogene in a variety of tumors, but its regulation methods are diverse. In cervical adenocarcinoma and bladder cancer, ALDOA affects tumor progression by regulating EMT process^9,36^. The interaction between ALDOA and ncRNA regulates tumor progression, a major research direction^10,11,32^. The discovery of the role of ALDOA in exosomes is a new direction in the field of ALDOA research^38^. At present, there are still few studies on ALDOA in tumors, and different ways of action are still being explored. Therefore, we hope to provide a general direction for future research through the pan-cancer analysis of ALDOA.

TNFSF4, also known as OX-40L, is a member of the TNF superfamily, which provides signals for CD4 T cell responses and plays an important role in tumor immunotherapy^24,39,40^. At present, the research of TNFSF4 mainly focuses on the immunomodulatory function in tumors and immune-related diseases^41^. Our results showed that ALDOA regulates the co-expression of TNFSF4 in a variety of tumors, which may have a certain correlation with the tumor’s immune microenvironment and prognosis. Other immune checkpoints in different tumors, and their relationship with ALDOA are also worth in-depth investigation. Perhaps there is a certain connection between glycolysis and tumor immunity, which needs to be further explored by other researchers.

Here, we describe the prognostic value of ALDOA in pan-cancer and report its immunological correlation with different cancers. As mentioned earlier, ALDOA is closely related to tumor metabolism, which plays vital roles in the tumor immune microenvironment^42^, but the relationship between ALDOA and tumor immune infiltration has not been clearly reported. We are surprised to find that there is a clear correlation between the two in LIHC, LUSC, BRCA and SKCM. However, it is far from enough to draw such a conclusion based on data analysis, and it needs to be verified by experiments in the future research. This study may provide new ideas for subsequent research and build a new relationship between metabolism and immunity.

## Conclusions

Based on our study, the expression level of ALDOA in most tumors is higher than that in normal tissues. Increased expression of ALDOA often indicates a poor prognosis for patients. Differences in the correlation between ALDOA and immune infiltration among different tumors was observed. For example, there is a positive correlation between the expression of ALDOA and immune infiltration in LIHC. However, there is a negative correlation in LUSC, BRCA and SKCM. At the same time, we found that ALDOA may be co-expressed with TNFSF4 to regulate tumor immune infiltration. Finally, we made a statistical analysis of the expression of ALDOA-related genes in pan-cancer. As far as we know, this is the first report on the correlation between ALDOA and tumor immune infiltration.

Based on this comprehensive analysis, we believed that ALDOA can be used as a prognosis biomarker in pan-cancer and is related to immune infiltration. Further studies can be performed to elucidate functions and detailed molecular mechanisms of ALDOA in tumor metabolism and immune microenvironment.

## Materials and methods

### Gene expression analysis

We used the ONCOMINE database (www.oncomine.org) to analyze the mRNA expression of ALDOA in different types of cancer. The significant expression of ALDOA between tumors and normal was recorded with a *P* < 0.001 and the fold change to 1.5.

### Survival prognosis analysis

We first analyzed the relationship between ALDOA and patient prognosis through PrognoScan (http://dna00.bio.kyutech.ac.jp/PrognoScan/index.html)^43^. We collected information about overall survival (OS), disease-free survival (DFS), relapse-free survival (RFS), distant metastasis-free survival (DMFS) and disease-specific survival (DSS). We followed the methods of Qingchen Yuan et al. 2020. and set the threshold to Cox *P-*value < 0.05^44^. Then we input tumor information from TCGA database and normal sample information from TCGA and GTEX project in GEPIA2 (http://gepia2.cancer-pku.cn/)^45^ to investigate the effect of ALDOA expression on OS and DFS in various tumors (n = 33). We also used Kaplan–Meier Plotter (https://kmplot.com/analysis/)^46^ to complement with above analysis. We calculate the hazard ratios (HRs) and log-rank *P* values with 95% confidence intervals (CI).

### Immune infiltration analysis

We searched ALDOA on the TIMER2.0 database (http://timer.cistrome.org/) to compare its relationship with immune infiltration in different tumors or specific tumor subtypes. We also selected cancer-associated fibroblasts for further analysis. At the same time, in order to perform reliable immune correlation assessment, we used immuneeconv, which is an R software package that integrates six latest algorithms, including TIMER, MCP-counter, xCell, EPIC, CIBERSORT and quanTIseq^47^. Perform a rank sum test on the data, and consider that *P* value of < 0.05 is statistically significant. Plot these data as heatmaps and scatter plots.

### Mutual-exclusivity analysis between ALDOA and multiple-immune checkpoints

We searched ALDOA and 30 multiple-immune checkpoints in the cBioPortal (http://www.cbioportal.org) to obtain co-expression or mutual exclusion information between them. Then we selected the genes that are significantly related to ALDOA and organized them into a table.

### ALDOA-related gene or protein analysis

We followed the methods of Cui et al.^47^. We searched ALDOA in the String (https://www.string-db.org/) database and obtained the ALDOA binding protein that has been verified by experiments. Next, we searched ALDOA in the "Similar Gene Detection" module of GEPIA2, and obtained the top 100 targeted genes related to ALDOA. We also applied the “correlation analysis” module of GEPIA2 to perform a pairwise gene Pearson correlation analysis of ALDOA and selected genes. Moreover, we used the data obtained by the “Gene_Corr” module of TIMER2 to draw a heatmap of the correlation between the target gene and ALDOA expression in pan-cancer.

### Statistical analysis

T-test, fold change, and gene grade determine the *P* value generated in Oncomine. Kaplan–Meier plotter and GEPIA2 curve analyses were generated by calculating HR and logrank *P*. HR and Cox *P* values in PrognoScan are calculated using univariate Cox regression model. The correlation of gene expression is assessed using Spearman's correlation^44^. In general, we determined *P* < 0.05 represents a statistically significant.

## Supplementary Information

Supplementary Information.

## Data Availability

The data and materials can be obtained by contacting the corresponding author.
