# Supplementary material for: Bioinformatics analysis of the role of aldolase A in tumor prognosis and immunity
Source: Sci Rep. 2022 Jul 8;12:11632. doi: 10.1038/s41598-022-15866-4 (PMC9270404; doi:10.1038/s41598-022-15866-4)
Supplement: Supplementary file 1 — Supplementary Information. [file 41598_2022_15866_MOESM1_ESM.pdf]

Supplementary Information for

Bioinformatics analysis of the role of aldolase A in tumor prognosis and immunity

Wanjia Tian[1,2], Junying Zhou[1], Mengyu Chen[1], Luojie Qiu[1], Yike Li[3], Weiwei Zhang[1], Ruixia Guo[1], Ningjing Lei[3*], and Lei Chang[1*]

[1] Department of Obstetrics and Gynecology, The First Affiliated Hospital of Zhengzhou University, Zhengzhou University, Zhengzhou 450000, Henan, China.

[2] Academy of Medical Sciences of Zhengzhou University, Zhengzhou University, Zhengzhou 450000, Henan, China.

[3] School of Basic Medical Sciences, Zhengzhou University, Zhengzhou 450000, Henan, China.

[*] Corresponding author：

Lei Chang (fccchangl@zzu.edu.cn)

Department of Obstetrics and Gynecology, The First Affiliated Hospital of Zhengzhou University, Zhengzhou, Henan 450000, China;

Ningjing Lei (lnj717@zzu.edu.cn)

School of Basic Medical Sciences, Zhengzhou University, Zhengzhou 450000, Henan, China.

This PDF file includes:

Supplementary Figure 1, Supplementary Figure 2, Supplementary Figure 3, Supplementary Figure 4, Supplementary Table 1, Supplementary Table 2.

Supplementary Figure 1: The Proportional Hazards assumption of a Cox Regression in GEO dataset is tested by Schoenfeld residuals. Schoenfeld residuals test *p* value in (A) OS in brain cancer cohort GSE16581. (B) OS in skin cancer cohort GSE19234. (C, D) OS and RFS in lung adenocarcinoma cohort GSE31210. (E) OS in breast cancer cohort GSE9893. (F) DMFS in breast cancer cohort GSE2990. (G, H) DSS and RFS in breast cancer cohort GSE1456-GPL96.

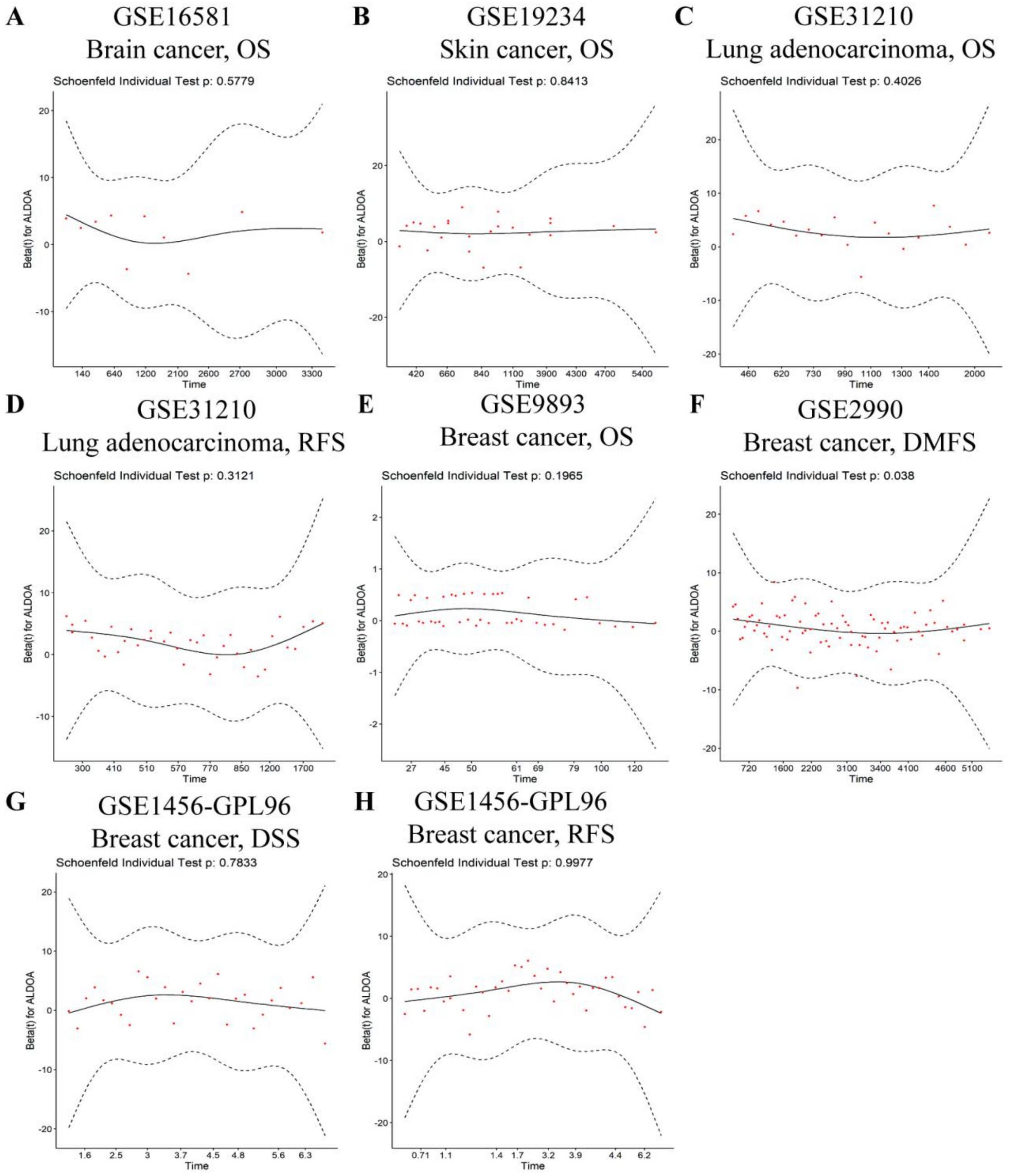

Supplementary Figure 2: The Proportional Hazards assumption of a Cox Regression in TCGA dataset is tested by Schoenfeld residuals. Schoenfeld residuals test *p* value in OS and RFS of **(A, B)** lung adenocarcinoma (LUAD) **(C, D)** cervical squamous cell carcinoma and endocervical adenocarcinoma (CESC) **(E, F)** thyroid carcinoma (THCA) **(G, H)** pancreatic ductal adenocarcinoma (PDAC) **(I, J)** head and neck squamous cell carcinoma (HNSC) **(K, L)** liver hepatocellular carcinoma (LIHC) **(M, N)** ovarian cancer (OC), and **(O, P)** kidney renal clear cell carcinoma (KIRC).

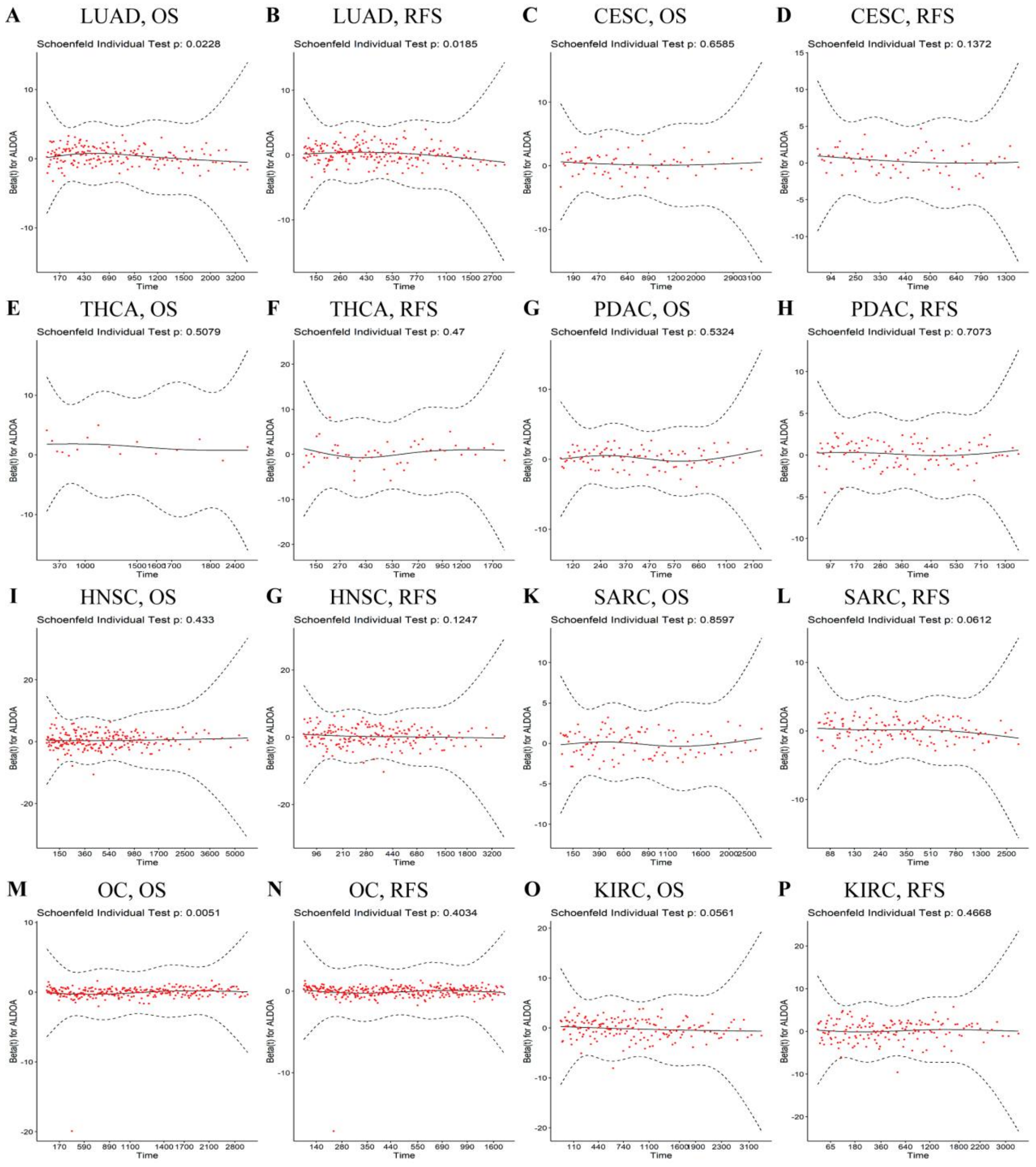

Supplementary Figure 3: Use GEPIA2 to collect the RNA sequencing data of ALDOA in TCGA, and analyze its influence on the prognosis of different tumors. Overall survival and disease free survival of (A) all cancer types (B) BLCA, Bladder Urothelial Carcinoma (C) CHOL, Cholangio carcinoma (D) GBM, Glioblastoma multiforme (E) LUSC, Lung squamous cell carcinoma (F) LUAD, Lung adenocarcinoma (G) LIHC, Liver hepatocellular carcinoma (H) TGCT, Testicular Germ Cell Tumors (I) CESC, Cervical squamous cell carcinoma and endocervical adenocarcinoma (J) SKCM, Skin Cutaneous Melanoma (K) LAML, Acute Myeloid Leukemia (L) THCA, Thyroid carcinoma (M) MESO, Mesothelioma (N) COAD, Colon adenocarcinoma (O) OC, Ovarian cancer (P) DLBC, Lymphoid Neoplasm Diffuse Large B-cell Lymphoma (Q) LGG, Brain Lower Grade Glioma (R) UVM, Uveal Melanoma (S) PRAD, Prostate adenocarcinoma (T) SARC, Sarcoma (U) BRCA, Breast invasive carcinoma (V) KIRP, Kidney renal papillary cell carcinoma (W) ACC, Adrenocortical carcinoma (X) KIRC, Kidney renal clear cell carcinoma (Y) KICH, Kidney Chromophobe (Z) ESCA, Esophageal carcinoma (AA) PCPG, Pheochromocytoma and Paraganglioma (AB) HNSC, Head and Neck squamous cell carcinoma (AC) STAD, Stomach adenocarcinoma (AD) THYM, Thymoma (AE) PAAD, Pancreatic adenocarcinoma (AF) READ, Rectum adenocarcinoma (AG) UCS, Uterine Carcinosarcoma (AH) UCEC, Uterine Corpus Endometrial Carcinoma.

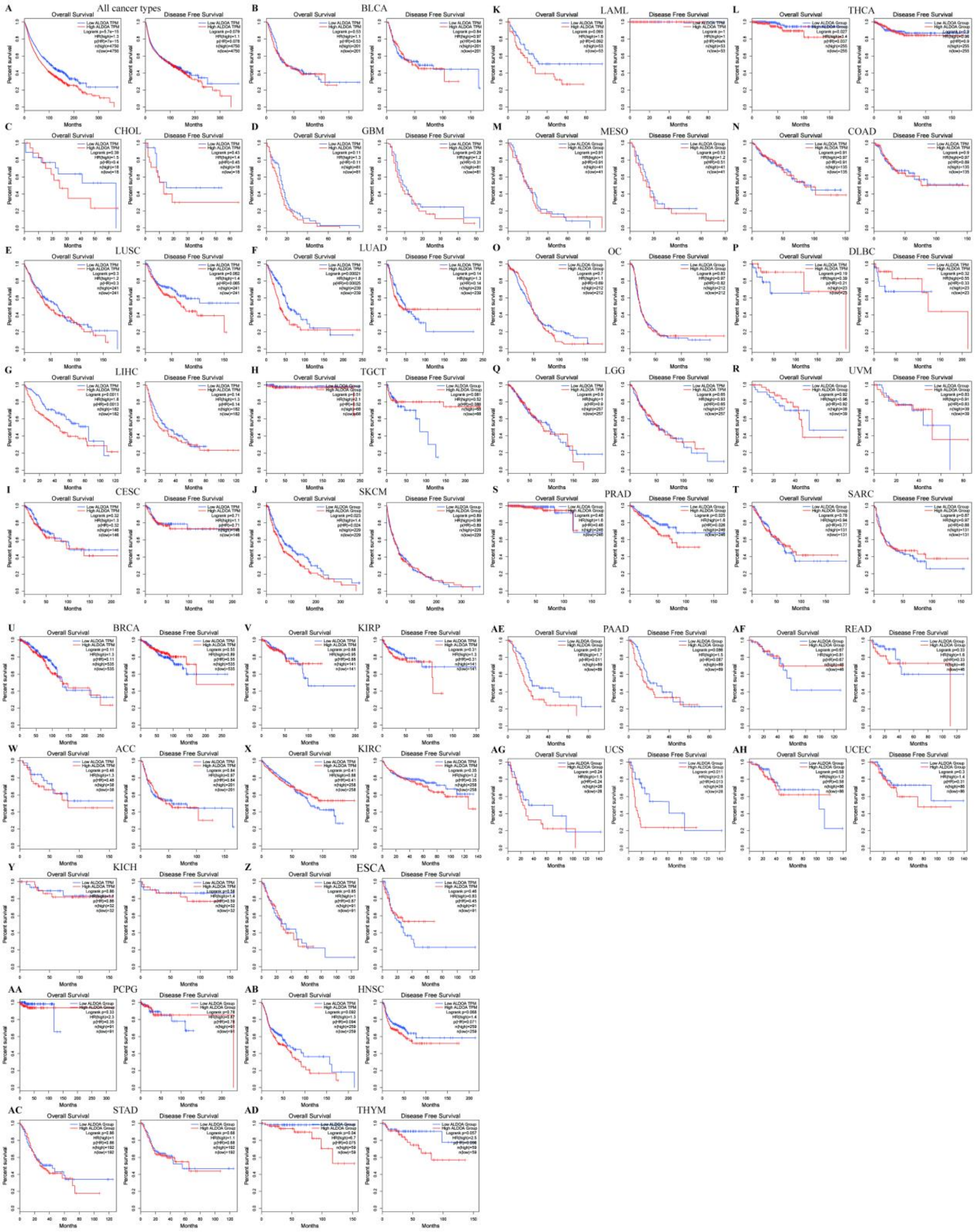

A All cancer types
B BLCA
C CHOL
D GBM
E LUSC
F LUAD
G LIHC
H TGCT
I CESC
J SKCM
K LAML
L THCA
M MESO
N COAD
O OC
P DLBC
Q LGG
R UVM
S PRAD
T SARC
U BRCA
V KIRP
W ACC
X KIRC
Y KICH
Z ESCA
AA PCPG
AB HNSC
AC STAD
AD THYM
AE PAAD
AF READ
AG UCS
AH UCEC
Overall Survival
Disease Free Survival
Percent survival
Months

Supplementary Figure 4: Use Kaplan-Meier Plotter to collect the RNA sequencing data of immune checkpoints in TCGA, and analyze its impact on the prognosis of LIHC and BRCA. The influence of (A) BTLA, (B) HHLA2, (C) CD27, (D) ICOS, (E) CD40, (F) ICOSLG, (G) CD40LG, (H) IDO1, (I) CD70, (J) LAFG3, (K) CD274, (L) NCR3, (M) CD276, (N) NT5E, (O) CTLA4, (P) PDCD1, (Q) ENTPD1, (R) PDCD1LG2, (S) FGL1, (T) SIGLEC15, (U) HAVCR2, (V) TMIGD2, (W) TNFRSF4, (X) TNFRSF9, (Y) TNFRSF18, (Z) TNFSF4, (AA) TNFSF9, (AB) TNFSF14, (AC) VSIR, (AD) VTCN1 on the OS and RFS of LIHC and BRCA.

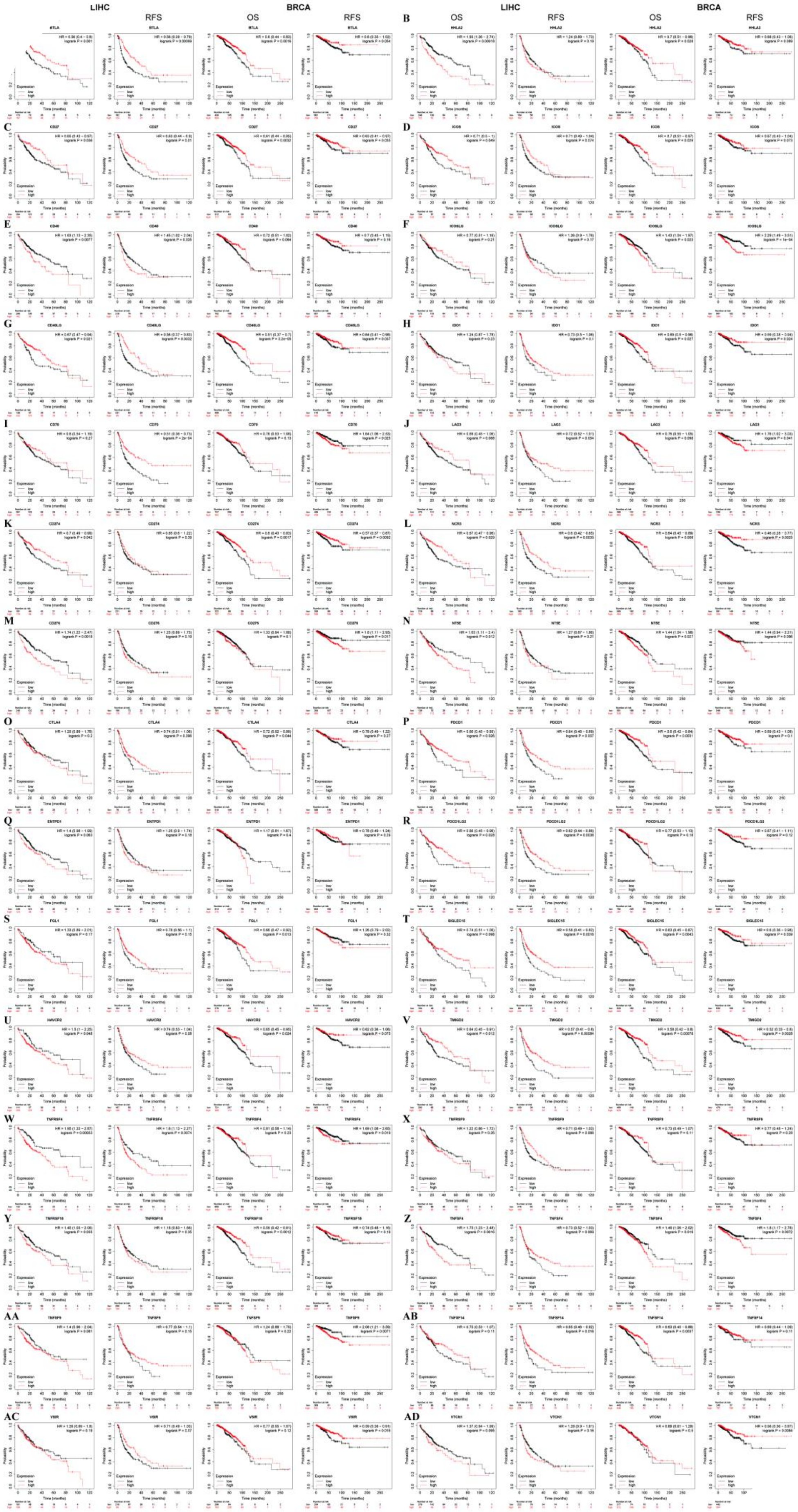
LIHC
RFS
BRCA
OS
RFS
B
OS
LIHC
RFS
BRCA
OS
RFS
C
D
E
F
G
H
I
J
K
L
M
N
O
P
Q
R
S
T
U
V
W
X
Y
Z
AA
AB
AC
AD

Supplementary Table 1: Accelerated Failure Time model was applied to further evaluate the association of the outcome and the expression of ALDOA.

GSE2990 Breast cancer DMFS	var	Estimate	StdErr	z.value	p.value
	ALDOA	-0.421	0.143	-2.943	0.003
LUAD OS	var	Estimate	StdErr	z.value	p.value
	ALDOA	-0.399	0.123	-3.249	0.001
LUAD RFS	var	Estimate	StdErr	z.value	p.value
	ALDOA	-0.235	0.092	-2.561	0.01
OC OS	var	Estimate	StdErr	z.value	p.value
	ALDOA	0.027	0.054	0.499	0.618

Supplementary Table 2: Mutual-exclusivity analysis between ALDOA and multiple-immune checkpoints in BRCA, LUAD and SKCM.

	A	B	Neither	A Not B	B Not A	Both	Log2 Odds Ratio	p-Value	q-Value	Tendency
BRCA	ALDOA	IDO1	3895	145	334	37	1.573	<0.001	<0.001	Co-occurrence
	ALDOA	VSIR	4161	168	68	14	2.35	<0.001	<0.001	Co-occurrence
	ALDOA	TNFSF4	3932	154	297	28	1.267	<0.001	<0.001	Co-occurrence
	ALDOA	NT5E	4188	177	41	5	1.529	0.039	0.076	Co-occurrence
	ALDOA	TNFSF9	4171	176	58	6	1.294	0.047	0.085	Co-occurrence
	ALDOA	CD276	4170	176	59	6	1.269	0.05	0.089	Co-occurrence
	ALDOA	NCR3	4182	177	47	5	1.33	0.062	0.106	Co-occurrence
	ALDOA	ENTPD1	4208	179	21	3	1.748	0.074	0.124	Co-occurrence
	ALDOA	TNFSF14	4162	176	67	6	1.083	0.079	0.131	Co-occurrence
	ALDOA	CD70	4161	176	68	6	1.061	0.083	0.136	Co-occurrence
	ALDOA	CD274	4136	181	93	1	-2.025	0.094	0.149	Mutual exclusivity
	ALDOA	PDCD1LG2	4138	181	91	1	-1.993	0.1	0.158	Mutual exclusivity
	ALDOA	TMIGD2	4157	176	72	6	0.977	0.101	0.159	Co-occurrence
	ALDOA	TNFRSF9	4176	182	53	0	<-3	0.106	0.163	Mutual exclusivity
	ALDOA	LAG3	4119	174	110	8	0.784	0.113	0.172	Co-occurrence
	ALDOA	SIGLEC15	4181	182	48	0	<-3	0.131	0.198	Mutual exclusivity
	ALDOA	CD40LG	4194	182	35	0	<-3	0.228	0.323	Mutual exclusivity
	ALDOA	VTCN1	4164	178	65	4	0.526	0.317	0.421	Co-occurrence
	ALDOA	FGL1	4062	176	167	6	-0.27	0.423	0.525	Mutual exclusivity
	ALDOA	ICOSLG	4159	180	70	2	-0.599	0.423	0.525	Mutual exclusivity
	ALDOA	CD40	4105	176	124	6	0.175	0.45	0.544	Co-occurrence
	ALDOA	TNFRSF18	4169	179	60	3	0.22	0.486	0.564	Co-occurrence

	ALDOA	CD27	4123	177	106	5	0.136	0.487	0.564	Co-occurrence
	ALDOA	TNFRSF4	4167	179	62	3	0.172	0.507	0.58	Co-occurrence
	ALDOA	BTLA	4187	180	42	2	0.148	0.548	0.602	Co-occurrence
	ALDOA	HHLA2	4172	180	57	2	-0.298	0.558	0.61	Mutual exclusivity
	ALDOA	CTLA4	4184	180	45	2	0.047	0.584	0.624	Co-occurrence
	ALDOA	HAVCR2	4196	181	33	1	-0.509	0.587	0.627	Mutual exclusivity
	ALDOA	PDCD1	4178	180	51	2	-0.136	0.625	0.649	Mutual exclusivity
	ALDOA	ICOS	4182	180	47	2	-0.016	0.671	0.687	Mutual exclusivity
LUAD	ALDOA	TNFSF4	1394	17	74	6	2.733	<0.001	0.014	Co-occurrence
	ALDOA	VTCN1	1434	19	34	4	>3	0.002	0.025	Co-occurrence
	ALDOA	NCR3	1435	20	33	3	2.705	0.016	0.121	Co-occurrence
	ALDOA	TNFSF14	1451	21	17	2	>3	0.033	0.206	Co-occurrence
	ALDOA	CD70	1457	22	11	1	2.59	0.171	0.61	Co-occurrence
	ALDOA	TNFSF9	1455	22	13	1	2.347	0.196	0.648	Co-occurrence
	ALDOA	VSIR	1455	22	13	1	2.347	0.196	0.648	Co-occurrence
	ALDOA	FGL1	1381	23	87	0	<-3	0.248	0.745	Mutual exclusivity
	ALDOA	SIGLEC15	1443	22	25	1	1.392	0.335	0.846	Co-occurrence
	ALDOA	NT5E	1442	22	26	1	1.334	0.345	0.847	Co-occurrence
	ALDOA	TMIGD2	1440	22	28	1	1.225	0.366	0.847	Co-occurrence
	ALDOA	CD27	1439	22	29	1	1.173	0.376	0.847	Co-occurrence
	ALDOA	LAG3	1432	22	36	1	0.854	0.441	0.849	Co-occurrence
	ALDOA	CD40LG	1425	23	43	0	<-3	0.508	0.849	Mutual exclusivity
	ALDOA	PDCD1LG2	1420	22	48	1	0.427	0.539	0.849	Co-occurrence
	ALDOA	CD274	1417	22	51	1	0.337	0.561	0.849	Co-occurrence
	ALDOA	HAVCR2	1432	23	36	0	<-3	0.568	0.849	Mutual exclusivity

	ALDOA	TNFRSF4	1433	23	35	0	<-3	0.577	0.849	Mutual exclusivity
	ALDOA	IDO1	1385	22	83	1	-0.399	0.625	0.849	Mutual exclusivity
	ALDOA	TNFRSF18	1439	23	29	0	<-3	0.634	0.849	Mutual exclusivity
	ALDOA	ENTPD1	1441	23	27	0	<-3	0.655	0.849	Mutual exclusivity
	ALDOA	TNFRSF9	1442	23	26	0	<-3	0.665	0.849	Mutual exclusivity
	ALDOA	PDCD1	1446	23	22	0	<-3	0.709	0.849	Mutual exclusivity
	ALDOA	ICOS	1448	23	20	0	<-3	0.731	0.851	Mutual exclusivity
	ALDOA	ICOSLG	1448	23	20	0	<-3	0.731	0.851	Mutual exclusivity
	ALDOA	CTLA4	1449	23	19	0	<-3	0.743	0.851	Mutual exclusivity
	ALDOA	CD40	1449	23	19	0	<-3	0.743	0.851	Mutual exclusivity
	ALDOA	CD276	1451	23	17	0	<-3	0.767	0.851	Mutual exclusivity
	ALDOA	HHLA2	1451	23	17	0	<-3	0.767	0.851	Mutual exclusivity
	ALDOA	BTLA	1455	23	13	0	<-3	0.816	0.861	Mutual exclusivity
SKCM	ALDOA	NCR3	1453	11	69	9	>3	<0.001	<0.001	Co-occurrence
	ALDOA	CD27	1494	14	28	6	>3	<0.001	<0.001	Co-occurrence
	ALDOA	NT5E	1474	14	48	6	>3	<0.001	<0.001	Co-occurrence
	ALDOA	CD276	1469	14	53	6	>3	<0.001	<0.001	Co-occurrence
	ALDOA	CD40LG	1510	17	12	3	>3	<0.001	0.006	Co-occurrence
	ALDOA	HHLA2	1425	14	97	6	2.654	0.001	0.01	Co-occurrence
	ALDOA	ICOSLG	1505	17	17	3	>3	0.002	0.012	Co-occurrence
	ALDOA	TNFRSF4	1482	16	40	4	>3	0.002	0.012	Co-occurrence
	ALDOA	TNFSF4	1481	16	41	4	>3	0.002	0.013	Co-occurrence
	ALDOA	FGL1	1481	16	41	4	>3	0.002	0.013	Co-occurrence
	ALDOA	TNFRSF9	1478	16	44	4	>3	0.003	0.015	Co-occurrence
	ALDOA	VTCN1	1477	16	45	4	>3	0.003	0.015	Co-occurrence

	ALDOA	TNFRSF18	1477	16	45	4	>3	0.003	0.015	Co-occurrence
	ALDOA	CTLA4	1501	17	21	3	>3	0.003	0.016	Co-occurrence
	ALDOA	ENTPD1	1466	16	56	4	2.71	0.006	0.026	Co-occurrence
	ALDOA	PDCD1	1459	16	63	4	2.533	0.009	0.036	Co-occurrence
	ALDOA	TMIGD2	1488	17	34	3	2.949	0.011	0.04	Co-occurrence
	ALDOA	SIGLEC15	1507	18	15	2	>3	0.019	0.059	Co-occurrence
	ALDOA	ICOS	1506	18	16	2	>3	0.022	0.063	Co-occurrence
	ALDOA	HAVCR2	1477	17	45	3	2.534	0.022	0.064	Co-occurrence
	ALDOA	IDO1	1436	16	86	4	2.062	0.026	0.071	Co-occurrence
	ALDOA	LAG3	1472	17	50	3	2.377	0.029	0.079	Co-occurrence
	ALDOA	CD40	1487	18	35	2	2.239	0.081	0.176	Co-occurrence
	ALDOA	CD70	1513	19	9	1	>3	0.123	0.228	Co-occurrence
	ALDOA	VSIR	1472	18	50	2	1.71	0.144	0.261	Co-occurrence
	ALDOA	BTLA	1504	19	18	1	2.137	0.221	0.365	Co-occurrence
	ALDOA	TNFSF9	1500	19	22	1	1.843	0.261	0.405	Co-occurrence
	ALDOA	TNFSF14	1480	20	42	0	<-3	0.574	0.668	Mutual exclusivity
	ALDOA	PDCD1LG2	1491	20	31	0	<-3	0.664	0.719	Mutual exclusivity
	ALDOA	CD274	1495	20	27	0	<-3	0.701	0.747	Mutual exclusivity
